# Equine Vaccines: How, When and Why? Report of the Vaccinology Session, French Equine Veterinarians Association, 2016, Reims

**DOI:** 10.3390/vaccines5040046

**Published:** 2017-12-04

**Authors:** Romain Paillot, Christel Marcillaud Pitel, Xavier D’Ablon, Stéphane Pronost

**Affiliations:** 1 Animal Health Trust (AHT), Lanwades Park, Kentford, Newmarket, Suffolk CB8 7UU, UK; 2 BIOTARGEN EA 7450, Normandie Université, UNICAEN—LABÉO, 14280 Saint Contest, France; stephane.pronost@laboratoire-labeo.fr; 3 RESPE (Réseau d’Epidémio-Surveillance en Pathologie Équine), 14280 Saint Contest, France; c.marcillaud-pitel@respe.net (C.M.P.); xavier.d-ablon@wanadoo.fr (X.D.); 4 Clinique Vétérinaire de la Côte Fleurie, Route de Paris—Bonneville sur Touques, 14800 Deauville, France

**Keywords:** vaccination, horse, outbreak, emergency vaccination, equine influenza, rhinopneumonitis, tetanus

## Abstract

To date, vaccination is one of the most efficient methods of prevention against equine infectious diseases. The vaccinology session, which was organised during the annual meeting of the French Equine Veterinarians Association (AVEF) at Reims (France) in 2016, aimed to approach three subjects of importance for the equine industry. Vaccination against three major equine diseases were used as examples: equine influenza (equine influenza virus), rhinopneumonitis (equine herpes virus 1/4), and tetanus (*Clostridium tetani* neuro-toxin). (1) Emergency vaccination: while it has been very successful to reduce the impact of equine influenza epizooties and it is also recommended for tetanus in case of surgery and accident, the benefit of emergency vaccination against equine herpes virus 1/4 remains arguable; (2) Compatibility of equine vaccines from different brands: despite being a frequent concerns for equine veterinarians, little information is available about the compatibility of equine vaccines from different commercial origins. The consequence of mixing different equine vaccines targeting the same disease is believed to be limited but scientific evidences are sparse; and, (3) Laps vaccination and vaccine shortage: they could have serious consequences in terms of protection and their impact should be evaluated on a case by case basis, taking into account the risk of contact with the pathogen and the effect on herd immunity.

## 1. Introduction

In many countries, the equine industry has a substantial economic weight (e.g., around £3.45 bn and 85,000+ direct and indirect employments linked to horse racing in the United Kingdom (UK) alone in 2013 [1]), represents a very large number of animals (e.g., >3.6 million horses in the United States of America (USA), 2.5 millions in Argentina, 0.8 to 1.2 million in the UK, France, and Germany each [2]). In this context, infectious diseases are major welfare and economic concerns, as illustrated in Australia in 2007 (major equine influenza epizooty with around 75,000 horses infected and an estimated cost to the Australian economy of A$1 bn reviewed in [3]). To date, vaccination remains one of the most efficient methods of prevention against several major equine infectious diseases. Most of the time, immunisation is simple and fast. However, the establishment of an efficacious and long lasting protective immunity is complex, both at the individual or herd levels. The vaccinology session, which was organised during the annual meeting of the French Equine Veterinarians Association (AVEF) at Reims (France) in 2016, aimed to approach three important questions: (1) what are the usefulness and efficacy of emergency vaccination; (2) what is the consequence of using/mixing vaccines from different brands and manufacturers; and, (3) what is the impact of vaccination laps or shortage on protective immunity. Three vaccines of importance for the equine industry were used as examples: equine influenza (equine influenza virus), rhinopneumonitis (equine herpes virus 1/4), and tetanus (*Clostridium tetani* (*C. tetani*) neuro-toxin).

## 2. What Are the Usefulness and Efficacy of Emergency Vaccination? 

In the face of an outbreak or an imminent threat, emergency vaccination could provide an important support to conventional biosecurity and prevention measures, such as movement restriction and/or interdiction, surveillance, and biosafety procedures. However, emergency vaccination should take into account the nature of the infectious risk (highly contagious pathogen or transmission limited to fomites and contact, for example) and the local situation and environment (population density etc.).

### 2.1. Equine Influenza (EI)

Equine influenza is a respiratory disease that is caused by the equine influenza A virus (EIV). Equine influenza vaccination has a proven record of efficacy in endemic regions or when implemented in an emergency in order to reduce epizooties frequency and propagation [4,5,6]. 

**Figure 1:**
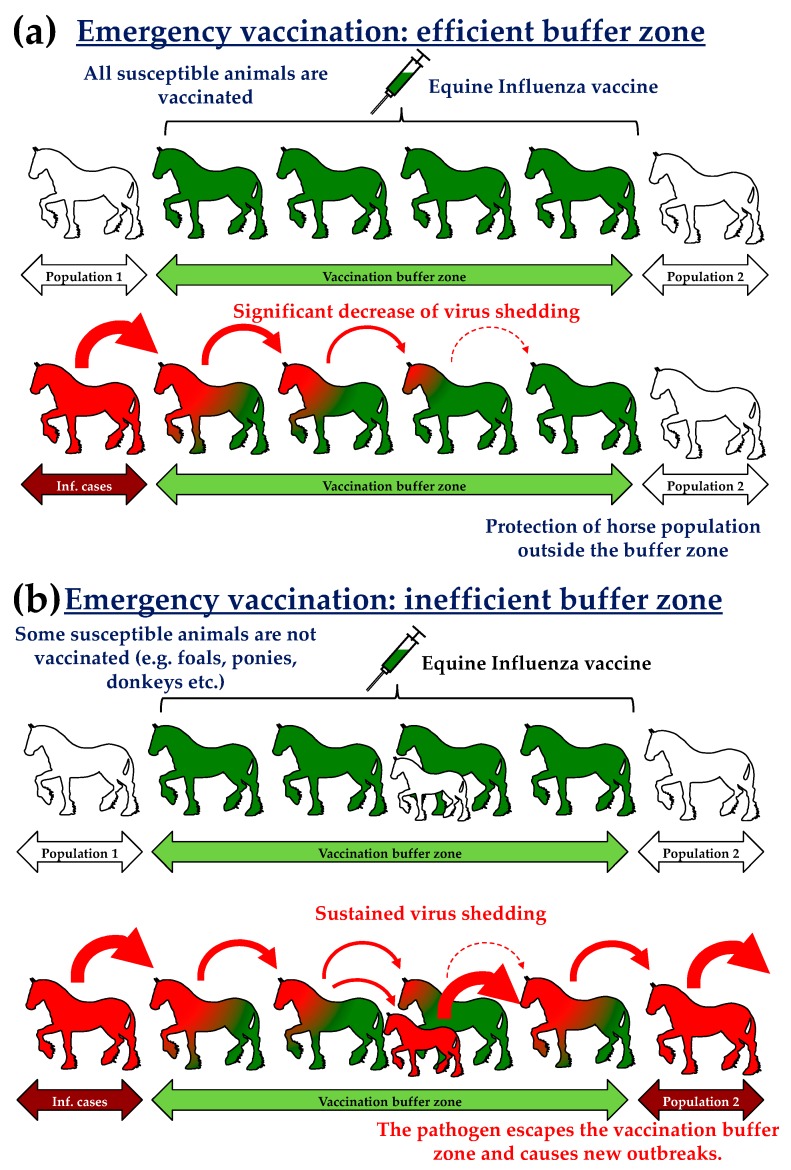
Emergency vaccination; (**a**) efficient or (**b**) inefficient vaccination buffer zone. The age to vaccinate foals against equine influenza A virus (EIV) is variable and will depend of the EI vaccine used (i.e., usually from 4 to 11 months of age for most Equine Influenza (EI) vaccines commercialized) and the presence of maternal-derived antibody (if the mare was vaccinated during pregnancy).

In 2007, a substantial EI epizooty had affected Australia. Around 75,000 equids were infected over a period of five months [3,4,5]. An emergency vaccination programme was implemented four weeks after the start of the outbreak and involved between 130,000 and 170,000 horses. The vaccination strategy involved several elements:

- The establishment of vaccination zones (10 km wide buffer zone; Figure 1) surrounding infectious areas (i.e., zones where infected horses have been detected). Subsequent epidemiological models and analysis revealed that the « suppressive » immunisation (i.e., 1 to 3 km radius) induced the best results with a marked reduction of new cases (−64%) and affected areas (−9%) [7,8]. The Australian state of Queensland was compartmentalised by vaccination corridors separated from each other by 25 to 35 km. Buffer zones were also used to protect 5 to 8000 wild equids in the state of New South Wales in order to avoid an endemic establishment of the disease. By the end of November, between 80% and 90% of horses in Queensland and New South Wales were vaccinated. Such levels of vaccination were above the levels usually recognised and described to provide herd immunity against infection [9].
- A targeted vaccination (or predictive) limited to specific horse populations of strategic and/or economic importance (thoroughbreds, police horses, competition horses, as examples).
- In some regions, vaccination was mandatory.

**Figure 2:**
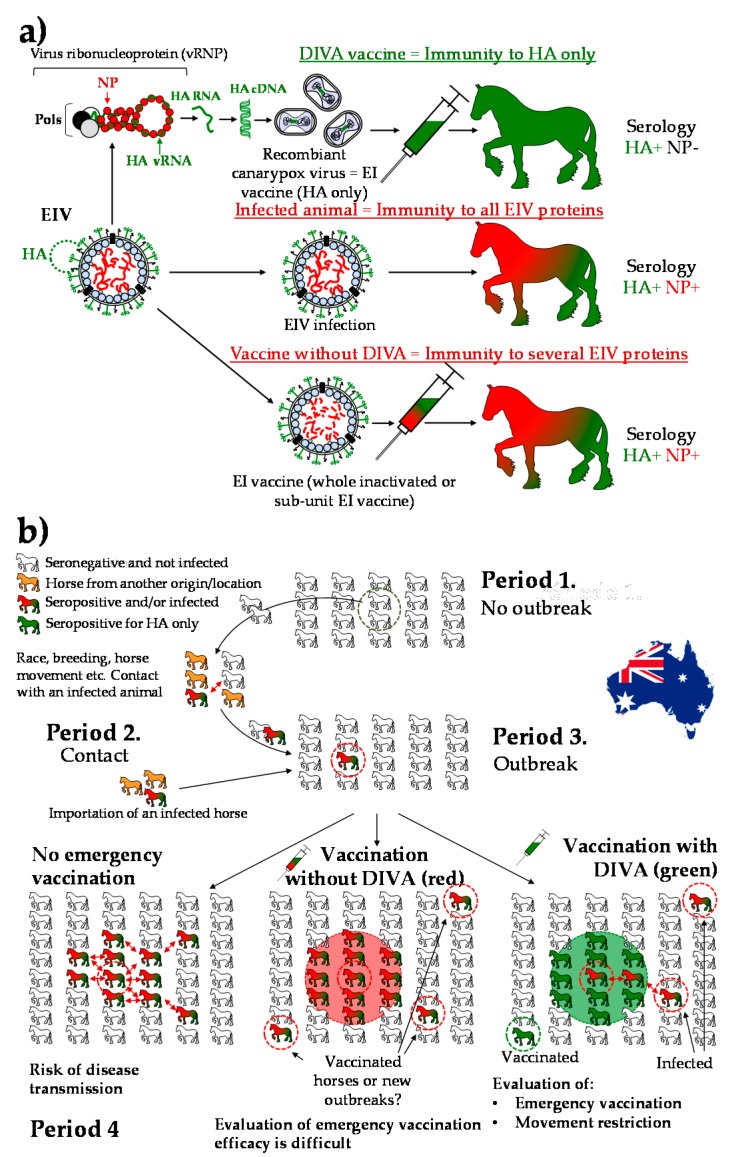
The importance of Differentiating Infected from Vaccinated Animals (DIVA). The use of an EI vaccine with DIVA capability is an asset to maintain an effective surveillance during an outbreak while emergency vaccination is implemented [3]. (**a**) The canarypox-based EI vaccine is a live attenuated canarypox virus with the EIV HA gene (green) inserted in its genome (one EIV HA per canarypox vector). The canarypox-based EI vaccine induces a seroconvertion limited to the EIV HA antigen after immunisation (green horse). Infection with EIV or immunization with whole inactivated or sub-unit EI vaccines induce a seroconvertion to several EIV antigens, including the EIV HA (green) and the nucleoprotein (NP, red) [12]; (**b**) An equid population naïve for EI (Period 1). Due to horse movement and/or importation of an infected animal (Period 2), an EI outbreak is detected (green + red horse, Period 3). Prevention and control measures are implemented (Period 4). In the absence of emergency EI vaccination, disease control relies primarily on movement restriction, active surveillance, biosecurity measures and is heavily dependent of the horse population density. A virus such as EIV is likely to spread quickly (especially in a naïve population such as in Australia in 2007). Emergency vaccination is implemented to support these measures. If an EI vaccine without DIVA capability is used (green + red vaccine), any seroconvertion (green + red horse) detected outside the vaccination buffer zone should be considered as a potential EI case (i.e., it is not possible to discriminate between a vaccinated horse that moved from the vaccination buffer zone or a new infected horse). The use of an EI vaccine with DIVA capability (green vaccine) allows to follow the spread of EIV infection inside the vaccination buffer zone, to identify real EI outbreak and infected horses (green + red horses) outside the vaccination buffer zone and to control the implementation of specific measures such as movement restriction.

It is quite well accepted and recognised that emergency vaccination has contributed to a reduction in EIV transmission in Australia (for both speed and distance of transmission). A significant reduction of clinical signs was also reported in vaccinated horses subsequently in contact with EIV (i.e., animals infected in the days following their first immunisation) [10,11]. The choice of an EI vaccine with DIVA (Differentiating Infected from Vaccinated Animals) capability was essential to the implementation of the EI vaccine strategy (Figure 2). 

### 2.2. Rhinopneumonitis and Secondary Forms of the Disease

Equine herpes virus type 1 and 4 infection induces a respiratory disease that could be associated with secondary forms, such as abortion in the pregnant mare or encephalomyelopathy (EHM). A few vaccines are available but their efficacies remain limited [13,14]. To date, there is no clear guideline about emergency vaccination against EHV-1 and 4, which remains a controversial subject. The HBLB (Horserace Betting Levy Board) code of practice and the EHV-1 consensus statement from the American College of Veterinary Internal Medicine (ACVIM) remain evasive about the vaccination of naive animals (not previously immunised) in the face of a potential infection with EHV-1 (mostly with suspected cases of EHM) [15,16]. This lack of information may be the result of reports that potentially link EHV-1 vaccination history and frequency with the development of neurological disease [17,18]. The safe use of EHV vaccine in the face of an outbreak was identified as one of the 11 goals for improved EHV vaccine design during the last Havemeyer workshop on EHV-1 [19]. The American Association of Equine Practitioners (AAEP) highlights the possibility to immunise animals that have not been exposed and/or new arrivals in order to stimulate an anamnestic response (in horses that have been previously vaccinated) [20]. 

### 2.3. Tetanus

Tetanus is a neuromuscular disease induced by the synthesis and excretion of the tetanus neurotoxin during *C. tetani* infection. Tetanus vaccination is highly recommended if horses sustain a wound and/or will undergo surgery. A boost vaccination is advised if their last tetanus immunisation dates from six months or more (the boost immunisation aims to induce a rapid anamnestic immune response). In the case of non-immunised animals, emergency vaccination and the administration of local and/or systemic tetanus antitoxin/antiserum is recommended. It is important to remember that both tetanus vaccination and tetanus antitoxin treatment only target *C. tetani* neurotoxin (i.e., neutralisation of the tetanus neurotoxin following synthesis or administration of neutralising antibodies). The site of infection (*C. tetani*) needs to be identified and adequately treated. 

## 3. What Is the Immunological Impact of Using/Mixing Vaccines from Different Brands and Manufacturers?

Horses are likely to be frequently and repeatedly immunized during their life. However, it is quite unlikely that an individual horse will receive the same vaccine/product against a specific pathogen during his lifetime (e.g., change of owners, veterinary practitioners etc.). The compatibility between vaccines targeting the same pathogen/antigen but commercialised by different manufacturers is a recurrent concern for equine veterinarians. Regretfully, little information is available on the immunological impact induced by mixing different vaccine brands.

### 3.1. Equine Influenza Vaccines

The question of EI vaccines compatibility is linked to the constant evolution of EIV (i.e., antigenic drift that induces increasing genetic and antigenic differences between vaccine and circulating strains) [21,22] and the availability of numerous vaccine technologies on the market [23,24]. The number of possible vaccine combinations is large, which makes it difficult to answer this question by conducting clinical trials alone. 

Information provided by pharmaceutical companies is often limited. However, Intervet International has conducted a vaccine compatibility study in the context of a patent registration [25]. This study demonstrated that a primary immunisation with the live attenuated EI vaccine Flu Avert IN (intra-nasal administration), followed four weeks later by a secondary immunisation with the EI vaccine Equilis Prequenza (sub-unit vaccine adjuvanted with ISCOM-matrix and administered intra-muscularly) induced a robust immune response that is superior to the one obtained after primary and secondary immunisations with the Flu Avert vaccine alone [25]. The technical information that is provided in this patent is regretfully limited. 

In the absence of experimental data, sero-epidemiological studies provide some information. Results from two sero-epidemiological studies are summarised here:

- Equine influenza outbreak in Newmarket (United Kingdom), 2003: the epidemiological analysis revealed that risk of infection with EIV was significantly reduced in animals with a mixed vaccine history. A whole inactivated EI vaccine adjuvanted with hydroxide aluminum seems to have provided limited protection during this outbreak, which in part explains the benefit of mixed vaccination (the limited efficacy induced by this vaccine was balanced when other EI vaccines were used) (Table 1) [26]. However, the time since last vaccination was one of the most important factor associated with risk of infection, explaining some of the counter-intuitive associations of infection with increased age and increased number of immunization/types of EI vaccines administered (i.e., ≥4 types in Table 1). Older horses (likely to have received multiple vaccinations and different types of EI vaccines) are likely to be immunized on an annual basis or to have lapse vaccination due to a lack of requirement. In this condition, the gap between immunisations received may be greater in older horses when compared to two-year old horses (identified as the less affected group of horses in the epidemiological study) that have recently completed their primary course of immunization (i.e., V1, V2, and V3; cf. Section 4). The limited number of horses in this specific category (i.e., ≥4 types) (*n* = 21) when compared with the other groups (*n* = 116, 161 and 103, respectively) may have also weakened the statistical analysis.
- Mandatory EI vaccination in Hong Kong (preliminary result from a current study): around 30% of the horse population in Hong Kong is renewed every year (through importation). Equine influenza vaccination is mandatory for horses that are being exported to Hong Kong (in the weeks prior to exportation). Upon arrival, horses receive a fresh primary course of EI immunization with a unique EI vaccine. Due to numerous countries of origin and the diversity of EI vaccines used worldwide, the mix of EI vaccine in recently imported Hong Kong horses is inevitable. Preliminary results demonstrate that differences between pre- and post-importation EI vaccines has no measurable impact on EIV-specific SRH antibody response [27].

### 3.2. Rhinopneumonitis Vaccines (Equine Herpes Virus Type 1 and 4)

Several rhinopneumonitis vaccines are commercially available. EHV-1 and EHV-4 genetic and antigenic variability is believed to be relatively limited (i.e., around one point mutation involving single nucleotide substitution per 1000 bp between the EHV-1 strains Ab4 and V592) [28]. In this context, antigenic difference between EHV vaccines have probably a limited impact, for vaccines of similar technology (whole inactivated EHV vaccine, for example). The question of EHV vaccine compatibility may exist when different vaccine technologies are commercially available (in Europe for example, with whole inactivated and live-attenuated EHV vaccines). To our knowledge, there is no study and/or results that provide answers to this specific question. In summary, EHV vaccine compatibility is supposed for vaccines of similar technology (there is no field or experimental evidence to contradict this assumption). However, differences in vaccination schedules may exist and the nature of the adjuvant that is contained in the vaccine should also be taken into account. This notion of compatibility between EHV vaccines remains unknown and untested. 

### 3.3. Tetanus Vaccines

Tetanus vaccines contain an inactivated form of the tetanus toxin (toxoid). There are no antigenic differences between commercialised tetanus vaccines. As a consequence, tetanus vaccines are compatible, from an immunological point of view. However, vaccination schedules may differ between vaccines and brands, depending if the tetanus vaccine is formulated alone or in combination with another target (e.g., equine influenza and tetanus vaccines). 

## 4. Lapse in Vaccination History and Vaccine Shortage 

**Figure 3:**
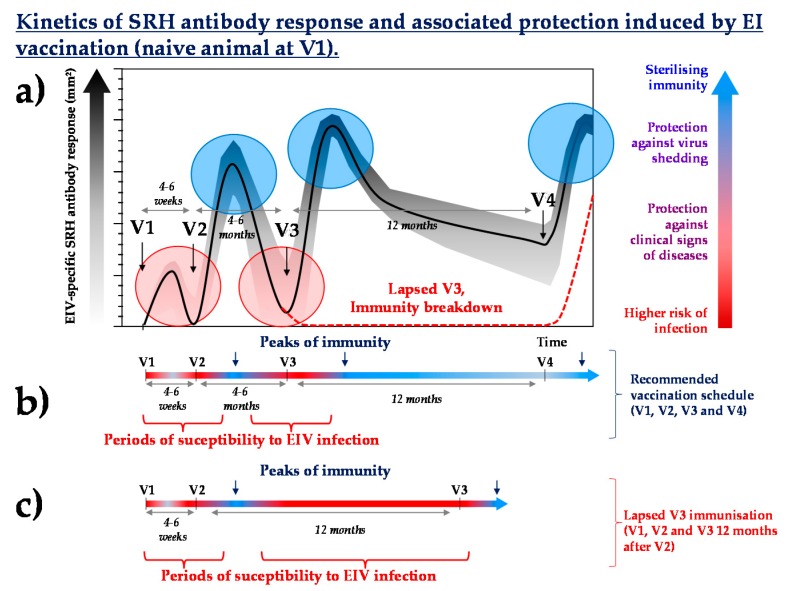
Kinetics schematic of the EIV-specific antibody response (**a**) induced by recommended vaccination schedules (**b**) ([29,30,31,32,33,34,35,36,37]) and potential impact of lapsed V3 immunisation (**c**). Blue circles represent usual peaks of immunity, red circles show usual periods of susceptibility to EIV infection. SRH = single radial haemolysis.

Lapsed vaccination happens when a vaccination schedule is not followed or maintained and in the case of a vaccine shortage. Lapsed vaccination should not be mistaken with immunity breakdown (often called “vaccination breakdown”), which corresponds to a default of immunity (i.e., disease occurrence) in an immunised animal (lapsed vaccination may result in immunity breakdown). For most vaccines and pathogen targets, the immunological consequences of lapsed vaccination depend on several key elements:

- What is the immunological history (vaccination and/or infection) of the individual? Lapsed vaccination could be a concern if it happens during the primary course of immunisation or shorty after. It is likely to be less problematic in adult horses that have been frequently and repeatedly vaccinated. For example, the absence of a third immunisation (V3) against EI (Figure 3) may increase the susceptibility to EIV infection by several months (until the next boost immunisation, usually scheduled 12 months after V3, 16 to 18 months after V2) and compromise the duration of immunity induced by the last vaccination.
- How efficacious and immunogenic is the vaccine used? Consequences of lapsed immunisation is likely to be inversely proportional to the duration of immunity of the last vaccine administered.
- What is the level of herd immunity? If the number of animals with lapsed immunisation is limited, then the risk of infection will be counterbalanced by the level of herd immunity of the surrounding population (i.e., the higher the herd immunity, the lower the risk of coming into contact with the pathogen).
- What is the risk of contact with the pathogen (i.e., isolated individual or numerous contacts with others equids)? Is there time for emergency vaccination? For most vaccines, reaching protective levels of immunity will take a few days only after boost immunisation if the horse has been frequently immunised (prior to lapse).

**Figure 4:**
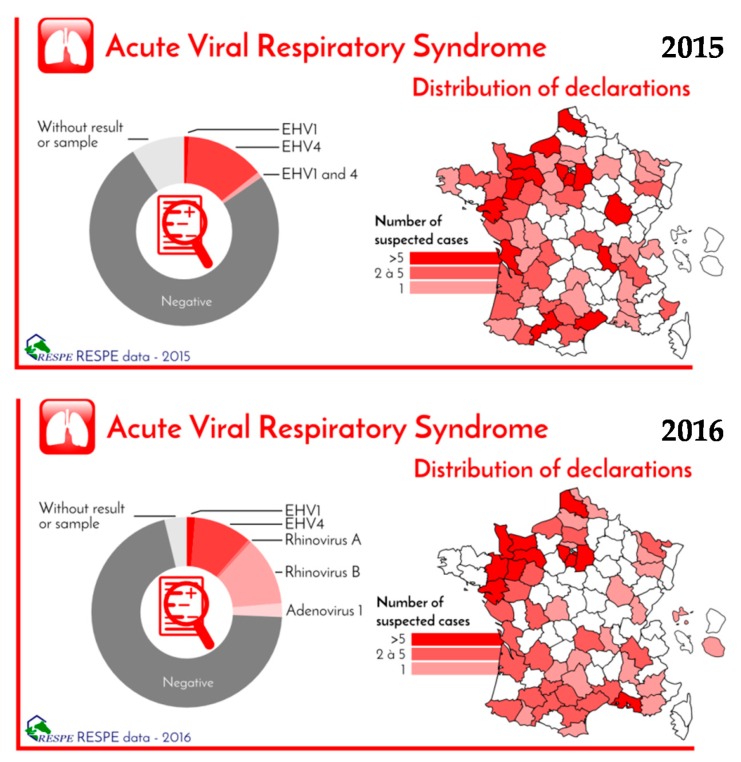
Equine acute viral respiratory disease reported to the French Epidemiological Surveillance of Equine Pathologies (RESPE) in 2015 and 2016. Causative agents and geographical localization are presented.

From 2015 to early 2017, numerous European countries have been affected with a shortage of rhinopneumonitis vaccines. The two veterinary pharmaceutical companies providing these vaccines in Europe (Merial Animal Health and Zoetis) experienced manufacturing issues with vaccine batch production and release, leading to a shortage of their rhinopneumonitis vaccines (Pneumequine ND and Equip EHV-1/4 ND, respectively). Both companies obtained temporary authorisation to import and commercialise substitute EHV-1 vaccines (Pneumabort K+1B produced by Zoetis in the USA and Bioequin H ND produced by Bioveta and supplied by Merial). The welfare impact has been limited, but consequences for the French equine industry and veterinary practitioners were important:

- In case of a vaccine shortage, veterinary practitioners have to face clients and horse owners discontent, which is justified by the concern of potential outbreaks (especially the risk of abortion in the case of EHV-1 infection). A vaccine shortage could induce a heavy financial loss (loss of commercial discounts and immobilisation of scheduled vaccine stocks).
- Both substitute products are monovalent EHV-1 vaccines. The number of doses available were limited (e.g., 150,000 doses of Bioequine H ND provided for 2016), which inevitably led numerous French veterinary clinics to prepare new stocks in order to cover the requirement. However, the use of these stockpiles of monovalent rhinopneumonitis vaccines was a concern, especially in case of the renewed availability of the bivalent EHV-1/4 vaccine.
- Financial loss also concerned horse owners, due to more expensive substitute vaccines, the absence of commercial discount for these new products and the necessity to administer new primary immunisation courses for horses with lapsed vaccination history (in order to comply with race and event regulations).
- The shortage of bivalent EHV-1/4 vaccine did not result in a notable increase of EHV-4 outbreaks being reported to the French Epidemiological Surveillance of Equine Pathologies (RESPE) between 2015 and 2016 (Figure 4). However, it had a negative impact on horse exportation due to the requirement for immunisation against both EHV-1 and EHV-4 in some countries and stud farms.

## 5. Conclusions 

In the case of EI, emergency vaccination could significantly reduce the size and frequency of epizooties, as clearly demonstrated in South Africa (2003) and Australia (2007). However, the use of an EI vaccine with a DIVA marker is highly recommended. Several vaccines will reduce virus shedding but sterilising immunity is rarely observed. In this case, vaccinated horses may be infectious (duration of virus shedding is likely to be reduced when compared with unvaccinated horses). In this context, the implementation of other preventive measures (such as biosecurity, movement restriction, surveillance, etc.) remains important. The usefulness of EHV emergency vaccination is more controversial, especially in naïve animals. In the case of tetanus immunization, emergency vaccination is essential to quickly neutralise the tetanus toxin.

The compatibility of vaccines from different brands and manufacturers is a frequent question from veterinary practitioners and horse owners. Concerning EI vaccines, the question is mostly focused around the EIV strains contained in the vaccines. To date, available data to evaluate the immunological impact of mixed vaccination (i.e., vaccination of the same animal with different EI vaccines that may contain different EIV strains) remain limited. In any doubt, more frequent boost immunisations (e.g., every 6 months) may be advocated, especially after a change of vaccine. Serological analysis may prove useful to prevent any default of immunity [37]. 

The immunological impact of lapsed immunisation or vaccine shortage is variable. The decrease in immunity is usually gradual with time passing since last immunisation (as opposed to the frequent public assumption of a sharp default of immunity). However, every breakdown in the vaccination schedule should be considered as potentially problematic, both at the individual and herd levels, and the consequences should be evaluated on a case-by-case basis. 

## Figures and Tables

**Table 1:** Impact of mixed EI vaccination during the 2003 EI outbreak in Newmarket (UK), from [26]. Significance was set at *p*-value ≤ 0.05 (bold text).

Variable	Category	EI Cases	Controls	Odds Ratio	*p*-Value
Number of EI vaccines administered	1 type	89 (76.7%)	27 (23.3%)	Reference	na
	2 types	110 (68.3%)	51 (31.7%)	0.65	0.13
	3 types	57 (55.3%)	46 (44.7%)	0.38	0.001
	≥4 types	15 (71.4%)	6 (28.6%)	0.76	0.6
Last EI vaccine administered	Whole inactivated, hydory-aluminium adjuvanted vaccine	193 (79.1%)	51 (20.9%)	Reference	na
	Other type	78 (49.7%)	79 (50.3%)	0.26	<0.001
